# Is increased male flower production a strategy for avoidance of predispersal seed predation in andromonoecious plants?

**DOI:** 10.1002/ece3.7468

**Published:** 2021-03-20

**Authors:** Gaku Kudo, Akari Shibata

**Affiliations:** ^1^ Faculty of Environmental Earth Science Hokkaido University Sapporo Japan

**Keywords:** alpine plants, andromonoecy, floral gender, phenology, pollen donor, seed predator, sex allocation

## Abstract

Floral gender in angiosperms often varies within and among populations. We conducted a field survey to test how predispersal seed predation affects sex allocation in an andromonoecious alpine herb *Peucedanum multivittatum*. We compared plant size, male and perfect flower production, fruit set, and seed predation rate over three years among nine populations inhabiting diverse snowmelt conditions in alpine meadows. Flowering period of individual populations varied from mid‐July to late August reflecting the snowmelt time. Although perfect flower and fruit productions increased with plant size, size dependency of male flower production was less clear. The number of male flowers was larger in the early‐flowering populations, while the number of perfect flowers increased in the late‐flowering populations. Thus, male‐biased sex allocation was common in the early‐flowering populations. Fruit‐set rates varied among populations and between years, irrespective of flowering period. Fruit‐set success of individual plants increased with perfect flower number, but independent of male flower number. Seed predation by lepidopteran larvae was intense in the early‐flowering populations, whereas predation damage was absent in the late‐flowering populations, reflecting the extent of phenological matching between flowering time of host plants and oviposition period of predator moths. Seed predation rate was independent of male and perfect flower numbers of individual plants. Thus, seed predation is a stochastic event in each population. There was a clear correlation between the proportion of male flowers and the intensity of seed predation among populations. These results suggest that male‐biased sex allocation could be a strategy to reduce seed predation damage but maintain the effort as a pollen donor under intensive seed predation.

## Graphical abstract

The proportion of male flowers increased with increasing risk of predispersal seed predation in an andromonoecious herb, *Peucedanum multivittatum*. This response might be a strategy to increase the fitness of pollen donors under intensive seed predation.

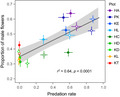

## 1 INTRODUCTION

Diverse sexual systems and gender expression in plants are thought to maximize reproductive success through pollen donation and seed production (Barrett & Harder, 2017). Sex allocation theory predicts that resource allocation to male and female functions is determined by the relationship between resource investment in a given sexual function and the fitness gain from that sexual function (Charlesworth, 1991; Charnov, 1982), which results in the evolution of diverse sexual systems. Many studies have reported phenotypic gender variation within and between populations of single species (Barrett, 2002; Charlesworth & Charlesworth, 1981; Lloyd, 1984). Various ecological factors, such as plant size, resource condition, selfing rate, pollinator availability, and plant architecture, affect sex allocation (reviewed in Barrett, 2002; Barrett & Harder, 2017). Furthermore, recent studies have reported that not only pollinators (mutualists) but also herbivores (antagonists) can affect the sexual system and phenotypic gender of plants (Clarke & Brody, 2015; Johnson et al., 2015; Wise & Hébert, 2010), although empirical studies demonstrating the significance of herbivory as an agent of selection on floral gender are limited (e.g., Wise & Hébert, 2010).

Of all types of herbivore damage, predispersal seed predation directly and intensively decreases the fitness of plants as it results in seed loss after resource investment in fruit development, which sometimes affects plant population dynamics (Ehrlén, 1996; Kolb et al., 2007; Maron & Crone, 2006). Plants have evolved various defense strategies against predispersal seed predation, such as the regulation of flower number, inflorescence size, flower color, flowering phenology, and floral volatile organic compounds (Elzinga et al., 2007; Johnson et al., 2015; Kolb et al., 2007; Rusman et al., 2019; Sercu et al., 2020; Valdés & Ehrlén, 2017). In particular, regulation of flowering phenology, that is, phenological avoidance, is often an effective avoidance strategy when seed predators have clear seasonal activity (Ehrlén, 2015; Pilson, 2000; Sercu et al., 2020; Valdés & Ehrlén, 2017); later flowering is commonly beneficial as it enables the plant to escape from predation damage (Ehrlén et al., 2015; Elzinga et al., 2007; Hendrix & Trapp, 1981; Mahoro, 2003; Miyake et al., 2018; Pilson, 2000). Because the intensity of predation damage varies spatiotemporally by a considerable amount (Ehrlén, 1996; Elzinga et al., 2007), floral trait adaptations may vary among neighboring populations of a single species, reflecting site‐specific interactions between plants and seed predators (Thompson & Cunningham, 2002).

Because of the sessile nature of plants, phenological events in plants are strongly influenced by abiotic factors within local habitats. This often restricts phenological regulation in plants responding to a selective force acting on specific phenological events, such as flowering and fruiting time. In snowy alpine ecosystems, lingering snow patches create mosaics of local environments in which the time of snowmelt strongly affects the growing period and reproductive schedule of alpine plants (Kudo, 1991). Because flowering time progresses sequentially along snowmelt gradients during the summer, alpine ecosystems provide opportunities to test the ecological significance of phenological variation on reproductive outcomes (Kudo, 2006). Previous studies revealed that pollination success and outcrossing rates in a single species varied greatly among neighboring populations along snowmelt gradients, reflecting the extent of phenological matching between flowering period and pollinator activity (Kameyama & Kudo, 2009; Kudo & Hirao, 2006; Kudo et al., 2011). The intensity of predispersal seed predation may also vary among neighboring populations along snowmelt gradients, but to date, no studies have investigated this possibility. In the present study, we aim to explore how intensity of predispersal seed predation varies seasonally and how alpine plants respond to the local variation in seed predation stress.

Several studies reported that herbivorous damages of flowers (florivory) affect floral gender and breeding system of plants (Ashman, 2002; McCall & Irwin, 2006; Wise & Cummins, 2007). In contrast to florivory that decreases the fitness through both male (pollen donation) and female (seed production) functions, predispersal seed predation (frugivory) intensively decreases the female fitness since frugivores consume developing fruits (Marshall & Ganders, 2001). Because a large amount of resources is wasted by predispersal seed predation, plants are expected to regulate reproductive allocation when the risk of seed predation is high. One possibility is a decrease in resource allocation to female function by which plants can reduce the predation damage during a reproductive event. On the other hand, plants may keep or increase the resource allocation to male function. When the seed predation rate is high and unpredictable in spatial terms, wide pollen dispersal may help to reduce the impact of predation of sired seeds. This theory is analogous to the colonization hypothesis of seed dispersal (Howe & Smallwood, 1982), which postulates that wider seed dispersal results in a higher probability that some seeds will encounter a safe site for survival and establishment.

In the present paper, we conducted a field survey to reveal how floral sex allocation varies among populations of single species under various seed predation damage. For this purpose, we selected an andromonoecious alpine herb, *Peucedanum multivittatum* (Apiaceae), that is a perennial iteroparous species with obligate outcrossing mating system. Andromonoecy, existence of male (staminate) and perfect (hermaphroditic) flowers within a plant, is a sexual system in which flexible regulation of resource allocation between female and male functions occurs (Lloyd & Bawa, 1984; Spalik, 1991). Male flowers sometimes show higher pollen dispersal ability than perfect flowers in andromonoecious plants (Dai & Galloway, 2012; Schlessman et al., 2004), and siring success often increases as male flower number increases (Elle & Meagher, 2000; but see also Tomaszewski et al., 2018). Therefore, we predicted that the proportion of male flowers would be higher in populations suffering from intensive seed predation than in populations in which such predation damage is less common.

The aim of this study was to detect the expected linkage between floral sex allocation and seed predation pressure in an andromonoecious species across local populations along natural snowmelt gradients. We compared gender expression, fruit‐set success, flowering phenology, and predispersal seed predation among local populations of *P. multivittatum* in areas where the timing of snowmelt differs. We compared both fruit‐set success and seed predation damage with respect to flowering phenology. Because pollinators and seed predators are counteracting selective agents (Altan et al., 2010; Elzinga et al., 2007; Johnson et al., 2015; McCall & Irwin, 2006), variation in sex allocation among populations needs to be interpreted in terms of both pollination success and avoidance of predation damage. The questions addressed in the present study are as follows: (a) How do fruit‐set rate and seed predation rate vary among local populations having different flowering phenology? (b) How does floral sex allocation, that is, the proportion of male flowers, vary among local populations along snowmelt gradients? (c) Is the pattern of floral gender variation related to fruit‐set success and/or risk of seed predation, or does it simply reflect plant size? Based on the results, we discuss the possibility that plants employ a sex allocation strategy to avoid predispersal seed predation.

## 2 MATERIALS AND METHODS

### 2.1 Plant material

**FIGURE 1:**
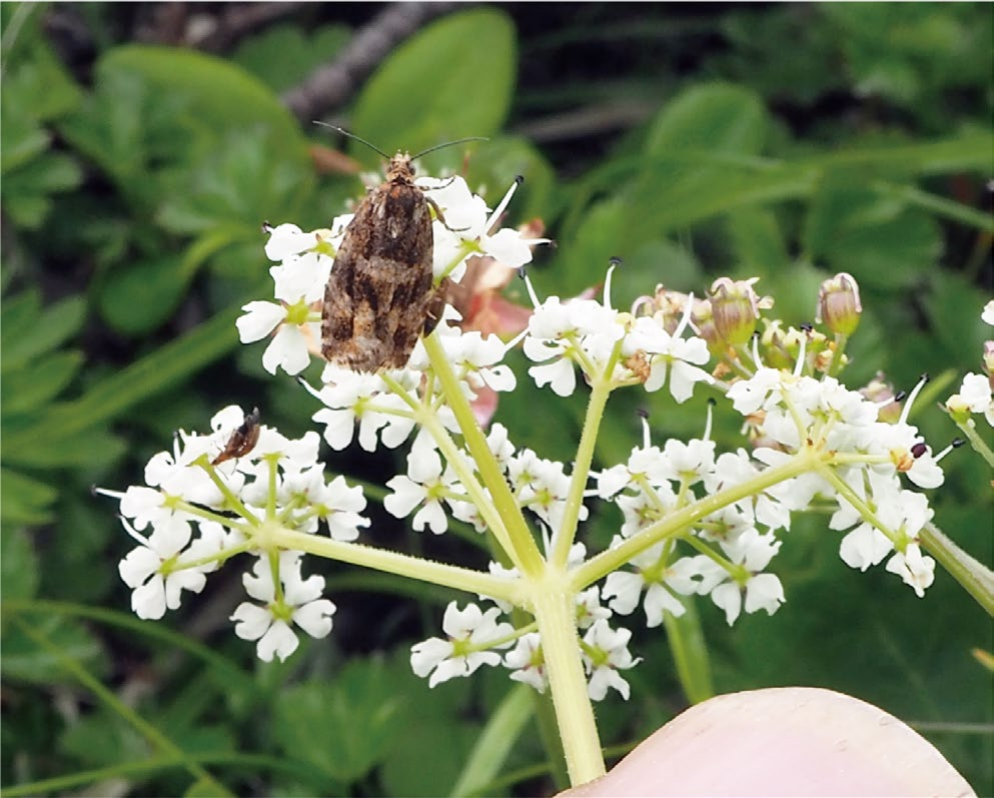
A predator moth (*Phaulernis fulvijuttella*, Epermeniidae) ovipositing on flowering peduncles of *Peucedanum multivittatum* (Apiaceae) in late July. Photograph by Gaku Kudo

*Peucedanum multivittatum* Maxim. (Apiaceae) is an alpine herb that inhabits snow meadows in alpine regions of Japan. Like many other apiaceous species, *P. multivittatum* is andromonoecious; it is characterized by a terminal umbel composed of male and perfect flowers with a few lateral umbels composed of only male flowers (Kudo, 1997). Protandrous flowering is common in perfect flowers, in which anthers dehisce soon after opening and pistils develop after the anthers are shed in the terminal umbel (Kudo, 1997). Flowering of male and perfect flowers occur simultaneously within a terminal umbel on which male flowers and perfect flowers at male phase are mostly identical in size and shape. The flowering of lateral umbels usually begins after the anthesis of the terminal umbel within a plant. Thus, dichogamous flowering, in which male and female phases occur at different times, is sequentially deployed within a plant. Flowering in *P. multivittatum* usually occurs between mid‐July and late August, depending on the time of snowmelt, and the flowering period within a population is about 10–14 days (Kudo, 1997; Kudo & Hirao, 2006). Fruits usually mature two weeks after flowering. This species is an obligate outcrosser, and major pollinators are dipteran insects, such as syrphids, and other flies. Developing seeds are often predated by specific fruit‐mining lepidopteran larvae (*Phaulernis fulvijuttella*; Epermeniidae). Our preliminary observation revealed that the predator moth deposited a few eggs on peduncles of terminal umbels during the flowering period (Figure 1), and major oviposition period was from mid‐ to late July in the study site.

### 2.2 Study site

This study was conducted in an alpine area of the Taisetsu Mountains, Hokkaido, northern Japan (43º32–33′N, 142º51–53′E). This mountain area is characterized by cold, snowy winters and mild, wet summers. The treeline is at around 1,500–1,600 m elevation. The annual mean temperature at 1,700 m elevation is −1.9ºC, ranging from −16.3ºC (January) to 12.5 ºC (August), and mean precipitation during the summer season (June to August) is 769 mm, ranging from 436 mm to 1,250 mm (in 2002–2019).

**FIGURE 2:**
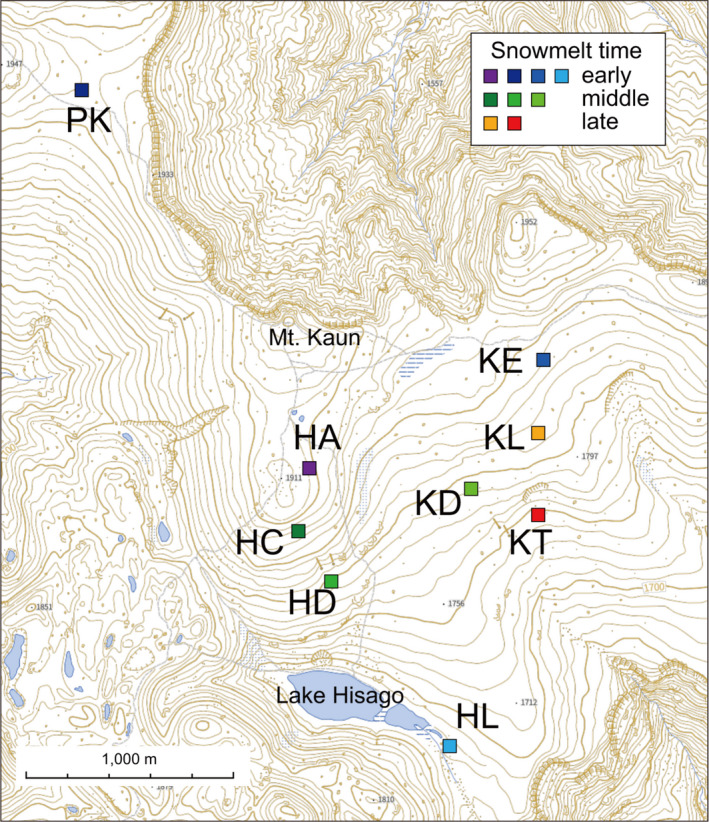
Location of research plots within the study area of the Taisetsu Mountains, northern Japan

We selected nine *P. multivittatum* populations in areas between 1,680 m and 1,915 m elevation where the timing of snowmelt differed: four early snowmelt populations (HA, PK, KE, and HL) in areas where the snow usually melts in early to mid‐June; three intermediate‐snowmelt populations (HC, HD, and KD) in areas where the snow usually melts in early to mid‐July; and two late‐snowmelt populations (KL and KT) in areas where snow usually melts in late July (Figure 2, Table 1). These populations were 230 m to 3,450 m apart each other. In each population, one 5 m × 20 m plot was set in the central part of the population so that it included at least 100 flowering individuals. This study was conducted over three flowering seasons from 2017 to 2019. Research in 2017 was conducted in five populations (HA, KE, HC, HD, and KL), and research in 2018 and 2019 was conducted in all nine populations.

**TABLE 1:** Snowmelt time and major flowering period in individual plots over 3 years

Plot	Elevation (m)	Snowmelt time			Major flowering period (DOY)		
		2017	2018	2019	2017	2018	2019
HA	1,900	Early Jun	Early Jun	Late May	191–201	197–205	189–203
PK	1,915	–	Mid‐Jun	Early Jun	–	199–206	191–204
KE	1,840	Mid‐Jun	Late Jun	Early Jun	196–206	201–209	189–203
HL	1,680	–	Mid‐Jun	Early Jun	–	200–207	193–206
HC	1,850	Mid‐Jul	Late‐Jun	Early Jul	213–226	200–207	210–221
HD	1,790	Late Jul	Mid‐Jul	Late Jul	217–235	213–222	222–235
KD	1,785	–	Mid‐Jul	Late Jul	–	215–225	222–235
KL	1,820	Late Jul	Late Jul	Late Jul	227–237	227–235	232–243
KT	1,750	–	Late Jul	Early Aug	–	227–235	237–251

### 2.3 Measurements in the field

In 2017, the effects of plant size on reproductive performance and floral gender were investigated in five populations (HA, KE, HC, HD, and KL). We randomly selected 40–50 flowering individuals in each plot during the flowering season and tagged selected plants with numbering tape. The flowering period in each plot was observed at 5‐ to 7‐day intervals. The major flowering period was defined as the duration from flowering initiation in 10% of plants to flowering termination in 90% of plants within a plot. During the flowering period, the basal diameter of the flowering stem, plant height, and the numbers of male and perfect flowers of marked plants were measured using a caliper and a ruler. Because basal diameter and plant height were highly correlated (Pearson's *r* = .67), basal diameter was used as an index of plant size in this study. At the young fruiting stage, before intensive predation damage, fruit number was recorded. Predated fruits were included in the fruit number because we aimed to evaluate pollination success. The fruit‐set rate of individual plants was expressed as the ratio of the number of fruits (irrespective of predation damage) to the number of perfect flowers. In the analyses of flower and fruit productions, only terminal umbels were targeted because lateral umbels are mostly composed of only male flowers and the production of lateral umbels is highly size‐dependent.

Surveys in 2018 and 2019 were conducted in all nine populations. In each plot, 40–50 individuals with flowering stems were arbitrarily selected and tagged at the floral bud stage, and the flowering phenology, number of male and perfect flowers, and number of developing fruits were recorded for each plant. All fruits were harvested after maturation and before dispersal, and the numbers of predated and intact fruits were recorded in the laboratory. The predation rate for individual plants was calculated as the ratio of the number of damaged fruits to the total number of fruits. We newly selected flowering individuals in each plot every year because about 70% of plants did not produce flowers in consecutive years, probably due to large resource investment in fruit production.

### 2.4 Statistical analysis

All statistical analyses were conducted using R version 3.3.3 (R Core Team, 2017). The size of flowering plants (basal diameter) was compared among five plots (HA, KE, HC, HD, and KD) using data from 2017 in a generalized linear model (GLM) postulating a Gamma error distribution with log‐link function, where plot was an explanatory variable. Then, size differences between individual plots were compared by Tukey's post hoc test at a significance level of *p* = .05 using the “multicomp” package. The effects of plant size on flower and fruit productions were tested using generalized linear mixed‐effects models (GLMMs) postulating a Poisson error distribution with log‐link function using the “lme4” package, where basal diameter was an explanatory variable and plot was included as a random factor. In the analysis, the numbers of total flowers, male flowers, perfect flowers, and fruits (irrespective of predation damage) on terminal umbels were analyzed separately. Furthermore, a relationship between male and perfect flower productions within umbels was analyzed by GLMM postulating a Poisson error distribution, where perfect flower number was a dependent variable, male flower number was an explanatory variable, and plot was a random factor. For each GLMM, pseudo‐*R* square value (*R* ^2^) was indicated to assess how dependent variable explained the model's variance using the “performance” package.

Flower production and floral gender (proportion of male flowers within an umbel) were compared across all plots using data from 2018 to 2019. The numbers of male and perfect flowers were compared among plots using a GLM postulating a Poisson error distribution, where plot and year were explanatory variables. For the GLM of floral gender, male flower number was a dependent variable, plot and year were explanatory variables, and total flower number was set as an offset term after logarithmic transformation. When significant differences were detected among populations, Tukey's post hoc test was performed between individual plots for pooled data across years.

Fruit‐set rate under natural conditions was compared among plots using data of 2018 and 2019 by a GLM postulating a Poisson error distribution, where fruit number (irrespective of predation damage) was a dependent variable, plot and year were explanatory variables, and perfect flower number was set as an offset term after logarithmic transformation. When significant differences were detected among populations, Tukey's post hoc test was performed between individual plots using pooled data across years. Furthermore, the effects of flowering time and floral gender on fruit‐set success were analyzed across plots and year using GLMM postulating a binomial error distribution with logit‐link function. In the GLMM, fruit‐set rate was a dependent variable, male flower number, perfect flower number, and flowering onset time in each plot were explanatory variables, and plot nested by year was set as a random factor. Flowering onset time means a period in which flowering started in each plot expressed by rank at 5‐day intervals since July 1, ranging from rank 1 (July 1–5) to 12 (August 26–30).

Predation damage to fruits was compared among plots and between years using a GLM postulating a negative binomial error distribution to reduce overdispersion because many zero values were included in the data. In this GLM, the number of damaged fruits was a dependent variable, plot and year were explanatory variables, and the number of fruits was an offset term after logarithmic transformation. When significant differences were detected among populations, Tukey's post hoc test was performed between individual plots using pooled data across years. Furthermore, the effects of flowering time and floral gender on seed predation rate were analyzed across plots and years using GLMM postulating a negative binomial error distribution using the “ADMB” package. In the GLMM, the number of damaged fruits was a dependent variable, male flower number, perfect flower number, and flowering onset time were explanatory variables, the number of fruits was an offset term after logarithmic transformation, and plot nested by year was set as a random factor.

## 3 RESULTS

### 3.1 Flowering phenology

**FIGURE 3:**
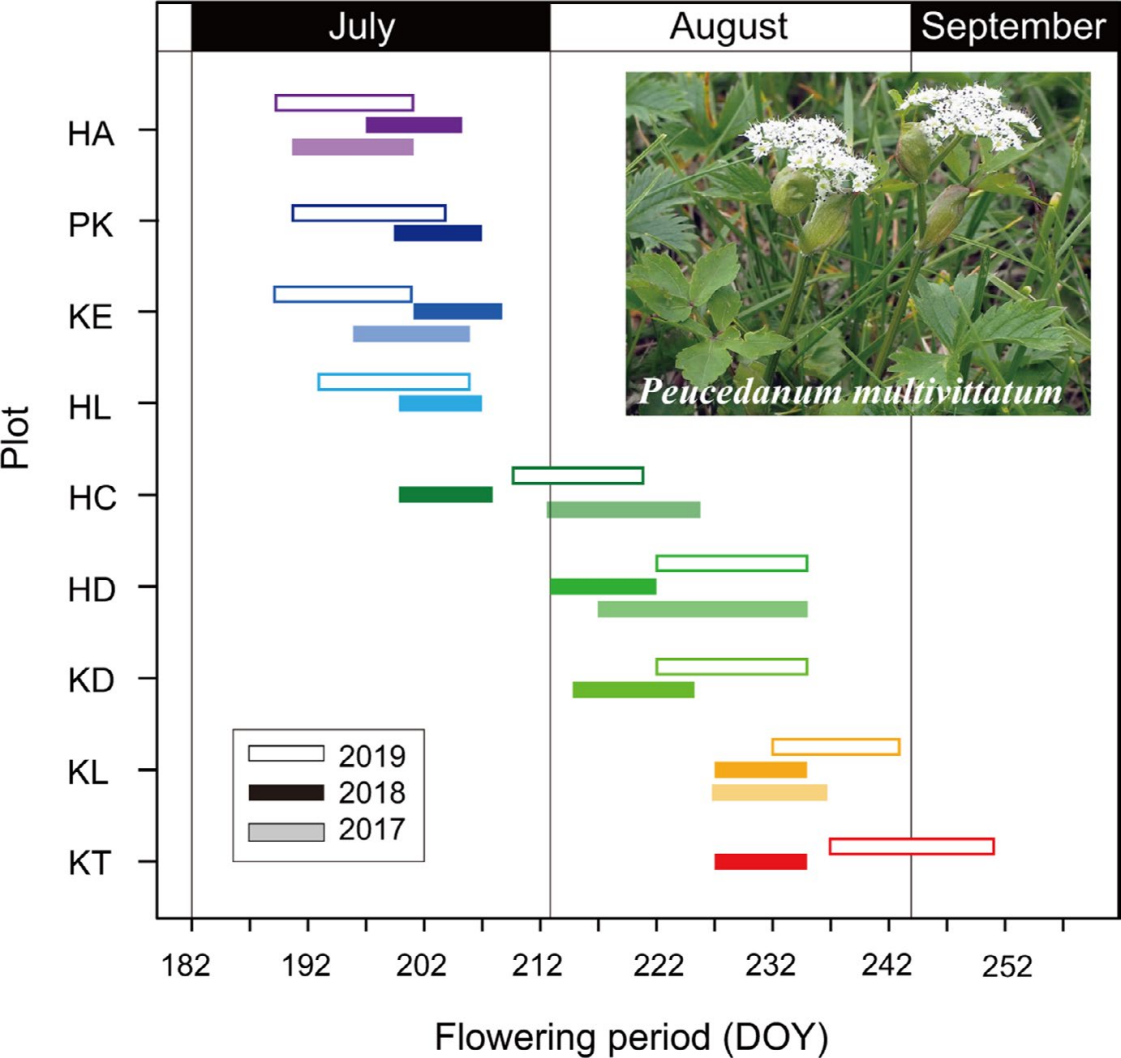
Major flowering period of *Peucedanum multivittatum* in each plot in each year (2017–2019). Measurement in 2017 was conducted for five of nine plots

The flowering period of *P. multivittatum* ranged from mid‐July to early September, reflecting the progress of snowmelt across populations (Figure 3, Table 1). Flowering in populations at early‐snowmelt plots (HA, PK, KE, HL) occurred around mid‐July in 2017 and 2019, while flowering in 2018 occurred in late July because snowmelt progressed slowly during June in that year. The major flowering period in populations at intermediate‐snowmelt plots (HC, HD, KD) was usually early to mid‐August. However, flowering in 2018 occurred earlier than usual, especially in HC, because snowmelt was accelerated after late June in that year. In populations at late‐snowmelt plots (KL, KT), flowering occurred after mid‐August due to very late snowmelt.

### 3.2 Effects of plant size on flower and fruit productions

**FIGURE 4:**
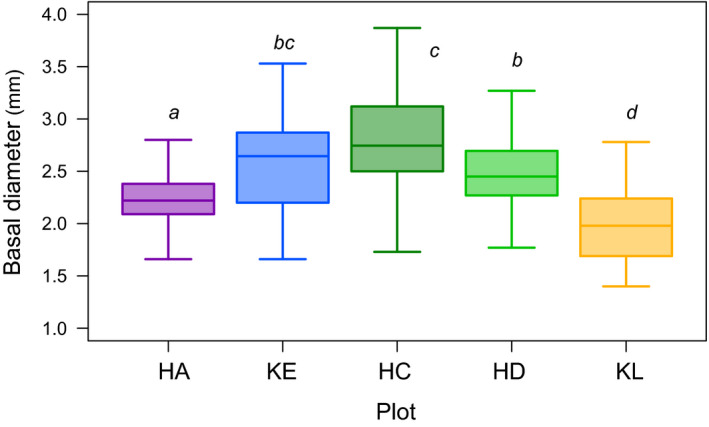
Comparison of basal stem diameter of *Peucedanum multivittatum*, as a representative of plant size, among five plots in 2017. Different letters indicate significant difference between populations by Tukey's test (*p* < .05). Boxes indicate quartiles, and whiskers show 1.5 times the interquartile range

**FIGURE 5:**
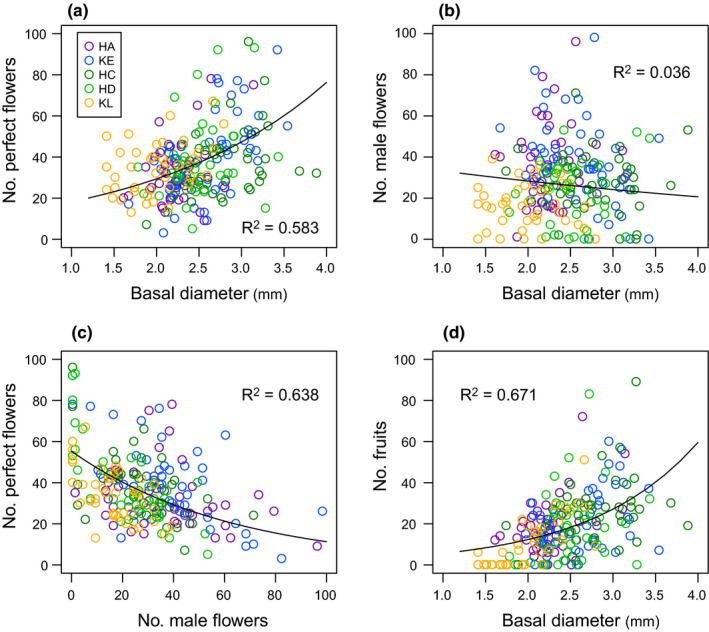
Size dependency of perfect flower production (a) and male flower production (b) per terminal umbel, relationship between male flower number and perfect flower number (c), and size dependency of fruit production (d) under natural pollination across five populations in 2017. Plant size is expressed by basal stem diameter. Regression line obtained by GLMM and pseudo‐*R* ^2^ value is shown in each relationship

Generalized linear models and multiple comparison tests revealed significant size differences among five populations (Figure 4). Plants at the intermediate‐snowmelt plot (HC) were largest, and plant size tended to be smaller at plots where snowmelt occurred earlier (HA) and later (KL) in the season. The number of perfect flowers per umbel increased with plant size (*z* = 16.42, *p* < .0001, *R* ^2^ = .58; Figure 5a). In contrast, the number of male flowers decreased with plant size (*z* = –4.68, *p* < .0001), but the decreasing pattern was gentle with large variance (*R* ^2^ = .036; Figure 5b), indicating that the size‐dependent variation in male flower production was less clear. As a result, total flower number, that is, sum of perfect and male flowers, increased with plant size (*z* = 9.74, *p* < .0001, *R* ^2^ = .26). There was a significant trade‐off relationship between male flower number and perfect flower number within terminal umbels (*z* = –21.97, *p* < .0001, *R* ^2^ = .64; Figure 5c). Fruit production increased with plant size (*z* = 20.11, *p* < .0001, *R* ^2^ = .67; Figure 5d), corresponding to the increased number of perfect flowers on larger plants.

### 3.3 Floral gender variation across populations

**FIGURE 6:**
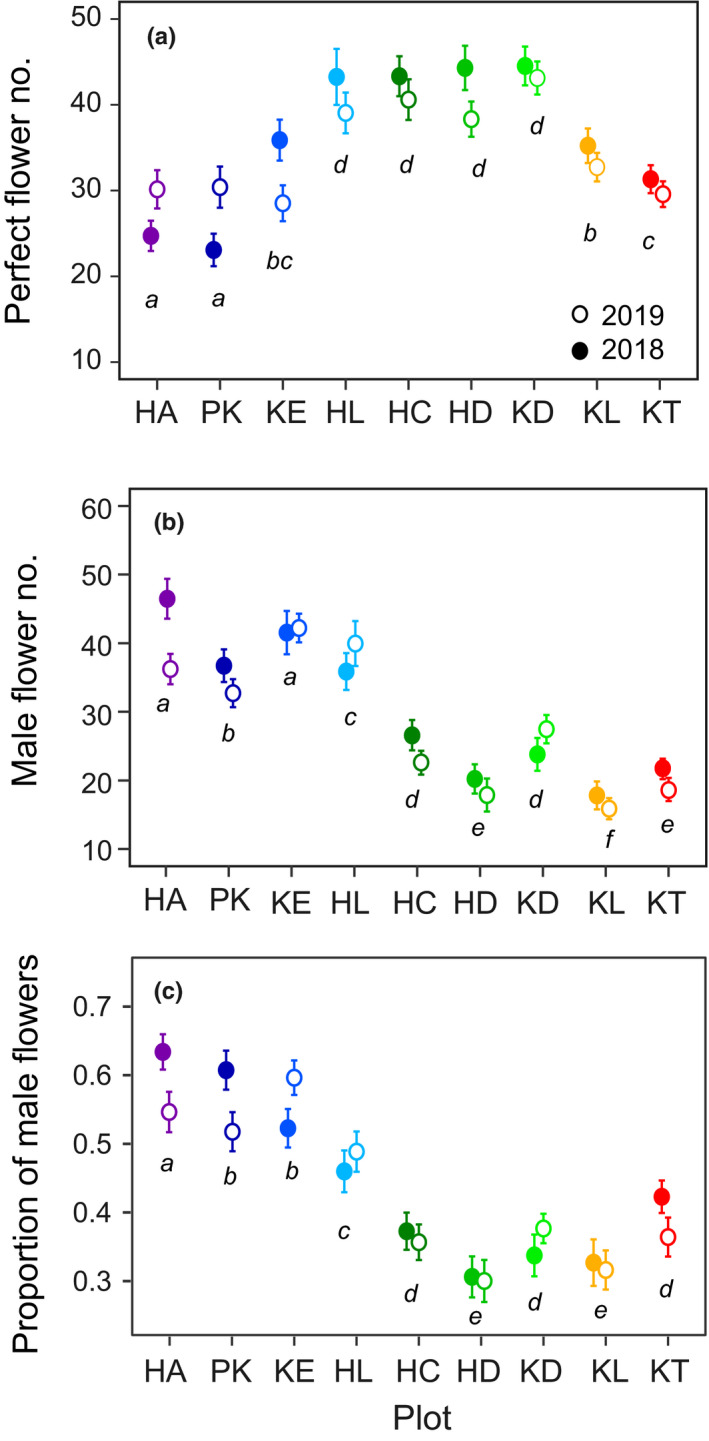
Comparisons of perfect flower number (a), male flower number (b), and proportion of male flowers on terminal umbels (c) among plots and between years. Different letters indicate significant differences between plots (*p* < .05, Tukey's post hoc test). Mean ± *SE*

There were significant differences in the numbers of male and perfect flowers per umbel among populations (Figure 6a,b). Plants at the early‐snowmelt plots (HA, PK, KE, HL) tended to produce more male flowers than those at the intermediate‐ and late‐snowmelt plots, whereas populations at the intermediate‐snowmelt plots (HC, HD, KD) produced many perfect flowers. Although significant yearly differences in flower production were detected by the GLMs (*p* < .01), the trend of flower production across the populations was similar between years. The proportion of male flowers at the early‐snowmelt plots (population mean in each year) ranged from 52% to 64% in HA, PK, and KE, and 46% to 49% in HL, while it was 30% to 43% in the middle‐ and late‐snowmelt plots (HC, HD, KD, KL, KT; Figure 6c). Thus, male‐biased flower production occurred consistently in the early‐snowmelt populations (Figure 6c).

### 3.4 Fruit‐set success across populations

**FIGURE 7:**
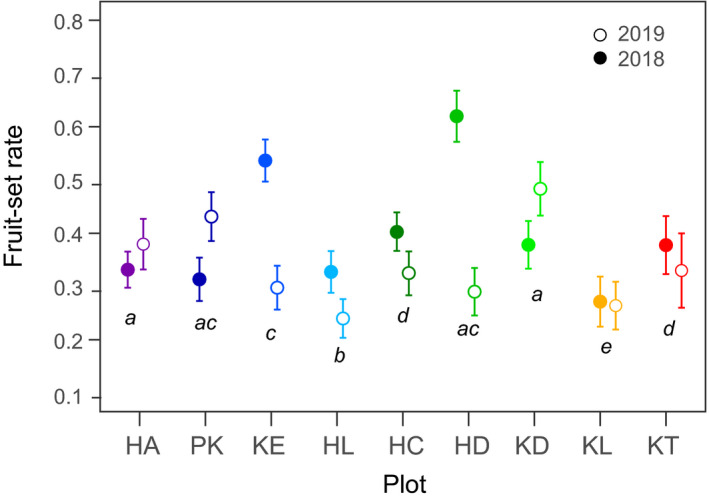
Comparison of fruit‐set rates under natural conditions among plots and between years. Different letters indicate significant differences between plots (*p* < .05, Tukey's post hoc test). Mean ± *SE*

The mean fruit‐set rate in each population ranged from 0.24 to 0.63 across plots and years. Although significant variation in fruit‐set rate was detected among plots (*p* < .001) and between years (*p* < .001) by the GLM, there was no consistent trend along the snowmelt gradient (Figure 7). The GLMM to test the factors affecting fruit production revealed that fruit‐set rate was positively related to the number of perfect flowers, whereas the numbers of male flowers and flowering time were independent of fruit‐set success (Table 2).

**TABLE 2:** GLMM result for the effects of perfect flower and male flower productions and flowering time on fruit‐set success

Variable	Estimate	*SE*	*z* value	*p* value
Intercept	−0.647	0.205	−3.15	**.0016**
Perfect flower no.	0.0078	0.0008	9.05	**<.0001**
Male flower no.	0.0001	0.0008	0.127	.90
Flowering time	−0.027	0.029	−0.97	.33

### 3.5 Effects of flowering time and gender variation on predispersal seed predation

**FIGURE 8:**
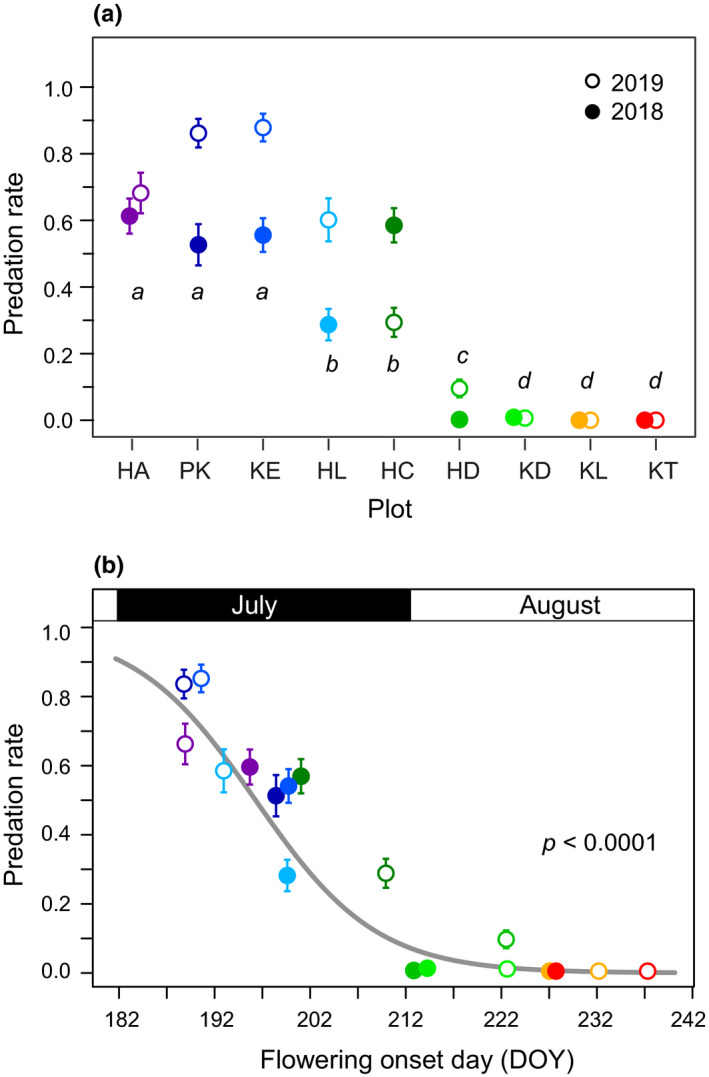
Comparison of predation rates of developing fruits among plots and between years (a), and the relationship between flowering onset time and predation rate shown by logistic regression (b). Different letters indicate significant differences between plots (*p* < .05, Tukey's post hoc test). Mean ± *SE*

There were significant differences in the predation damage to fruits among plots (*p* < .001) and between years (*p* < .001) by the GLM (Figure 8a). Predation damage was intense at the early‐snowmelt plots (HA, PK, KE, HL), ranging from 0.29 to 0.88, whereas there was no predation damage at the late‐snowmelt plots (KD, KL, KT). The GLMM to test factors affecting predation damage revealed that predation rate was negatively related to flowering time, while both male and perfect flower productions were independent of predation damage (Table 3). Plants suffered from high predation damage when flowering occurred before mid‐July, while predation damage was rare when flowering occurred after early August (Figure 8b). A similar trend was detected within a same population between years, that is, at the intermediate‐snowmelt plot HC, early flowering in 2018 (Figure 3) resulted in a higher rate of predation damage (59%) in comparison with the predation damage in 2019 (29%) when flowering occurred as usual (Figure 8).

**TABLE 3:** GLMM result for the effects of perfect flower and male flower productions and flowering time on seed predation damage

Variable	Estimate	*SE*	*z* value	*p* value
Intercept	1.787	0.631	2.83	**.0046**
Perfect flower no.	0.0029	0.0025	1.16	.24
Male flower no.	0.0011	0.0023	0.48	.63
Flowering time	−0.803	0.110	−7.29	**<.0001**

**FIGURE 9:**
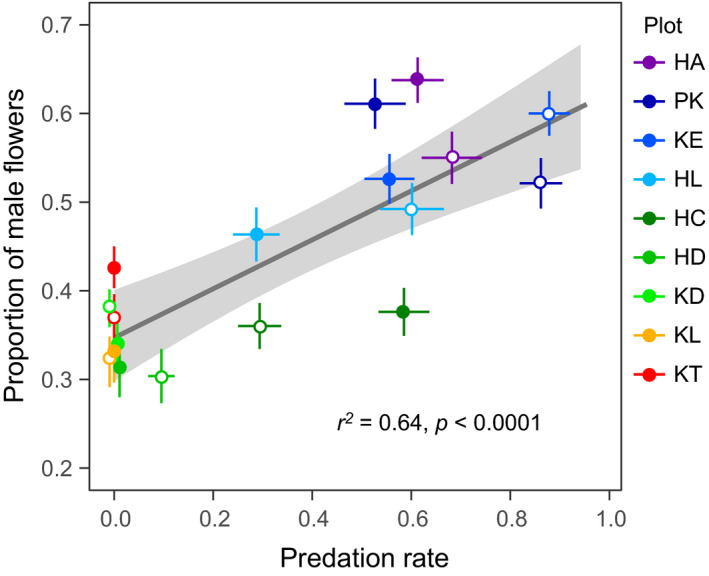
Relationships between seed predation rate and proportion of male flowers across populations. Mean ± *SE* of individual populations is shown. A regression line and 95% confidence level are indicated

There was a significant correlation between the risk of predation damage (mean predation rate in each plot) and the proportion of male flowers across populations and years (*r* ^2^ = .64, *p* < .0001; Figure 9). This indicates that the proportion of male flowers was higher in the populations suffering from continuous severe predation damage.

## 4 DISCUSSION

The major flowering period of individual populations ranged from mid‐July to late August, depending on snowmelt time, and seed predation damage was most intense in early‐snowmelt populations. Floral sex allocation varied significantly along the snowmelt gradient, and male flower production was positively related to the risk of seed predation at population level. These results support our prediction that male‐biased sex allocation is selected in andromonoecious species subject to intensive seed predation. To our knowledge, this is the first report of the linkage between floral sex allocation and predispersal seed predation among local populations.

Intensive seed predation in the early‐snowmelt populations was supposed to be caused by phenological matching between flowering in *P. multivittatum* and oviposition in *P. fulvijuttella*. Predatory moths oviposit on the peduncles of terminal umbels of host plants during the flowering period. Actually, moth's eggs were frequently observed in the early‐snowmelt populations but absent in the late‐snowmelt populations (personal observation). The predation rate was > 50% when flowering occurred before July 20, whereas little predation damage was observed when flowering occurred after July 30. These results indicate that the risk of seed predation is highest when flowering occurs in mid‐July. Although the actual flowering time of individual populations varied from year to year depending on the snowmelt time, the seasonal trend in predation damage was similar between years (Figure 8b), indicating a stable oviposition period between years. When predispersal seed predation is severe in early season, later flowering trait has evolved as a predator avoidance strategy in several species (Ehrlén, 2015; Pilson, 2000; Sercu et al., 2020; Valdés & Ehrlén, 2017). However, the regulation of flowering phenology may be less effective in alpine snow meadows because flowering time is strongly determined by snowmelt time and actual snowmelt time highly varies from year to year (Kudo, 2006). Actually, seed predation rates of HL and HC were two times higher in the year when snowmelt occurred earlier than usual years (Figure 8).

In contrast to the trend in predation damage, there was no clear trend in fruit‐set success among populations irrespective of large variation in flowering time. Major pollinators of this species are dipteran insects that are most common throughout the summer (Mizunaga & Kudo, 2017). Thus, difference in flowering period may be less important for pollination success in this species. Both total and perfect flower productions increased with plant size, and the number of perfect flowers was positively related to fruit‐set success. This means that plants having large umbels can have higher fruit‐set success, probably due to higher attraction of dipteran insects (Inouye et al., 2015). On the other hand, variation in floral sex allocation among populations was independent of pollination success.

Relationships between size and sex allocation have been reported previously in many animal‐pollinated plant species, where female‐biased sex allocation is common in larger plants (e.g., de Jong & Klinkhamer, 1989; Klinkhamer & de Jong, 1997). Although perfect flower production increased with plant size, the size dependency of male flower production was less clear in *P. multivittatum*. Plant size varied greatly among populations in which smaller‐sized plants were common in the earliest‐ (HA) and latest‐snowmelt (KL) populations. Small plant size at HA plot might be related to drier soil conditions due to early snowmelt. Meanwhile, small plant size at KL plot might be related to a very short growing season due to late snowmelt. Even when these marginal populations were compared, plants at HA plot were male‐biased, whereas plants at KL plot were female‐biased (Figure 6). Thus, the variation in floral sex allocation among local populations cannot be explained by plant size.

There are only a few studies on the relationship between floral gender and floral herbivory in andromonoecious plants. In two apiaceous species (*Heracleum lanatum* and *Pastinaca sativa*), for instance, herbivorous damage to first umbels resulted in increased production of perfect flowers in late‐developing umbels by the regulation of resource allocation within plants (Hendrix, 1984; Hendrix & Trapp, 1981). In another andromonoecious herb (*Solanum carolinense*; Solanaceae), the proportion of male flowers was negatively correlated with the intensity of weevil florivory among populations, because the production of many ovaries is important to maintain seed production under intensive florivorous conditions (Wise & Cummins, 2007). These compensative responses to floral herbivory indicate an opposite trend of sex allocation to that observed in the present study. This discrepancy means that populations are subject to different selective forces depending on whether they suffer from floral herbivory or predispersal seed predation. In the case of floral herbivory, resource investment in fruit development usually occurs after herbivory damage. In the case of seed predation, however, a large amount of resources has been invested in fruit development when seeds are predated; thus, compensative responses after seed predation may be difficult.

Predispersal seed predation results in an intensive reduction in female success in terms of seed production. The risk of seed predation was simply determined by flowering time of individual populations, and floral sex allocation of individual plants was independent of predation rate within a population (Table 3). It suggests that seed predation is a stochastic event in each population irrespective of floral gender, probably because predator moths do not discriminate the proportion of male flowers of individual umbels at the time of oviposition. When the risk of seed predation is high, plants may reduce the waste of resources due to seed predation by reducing fruit production during a single reproduction. As mentioned before, *P. multivittatum* is a perennial iteroparous species, and about 70% of fruiting plants do not produce flowers next season, indicating large cost of fruit production. By saving the resource investment in excess fruit production in each reproductive event, plants may be able to increase the frequency of reproductive events during the lifetime. Furthermore, plants can produce more male flowers at the expense of fruit production. Improvement of male success in terms of pollen donation could be a possible strategy against seed predation, that is, a spatial avoidance strategy by pollen dispersal. Although we did not evaluate the relationship between the number of male flowers and pollen donor success in this species, previous studies have reported that male flowers in andromonoecious plants have wider pollen dispersal and higher siring success than hermaphroditic flowers (Dai & Galloway, 2012; Elle & Meagher, 2000; Schlessman et al., 2004). Therefore, increased male flower production could be an effective avoidance strategy against intensive predispersal seed predation.

The present study suggests that predispersal seed predation can be a selective agent for floral sex allocation at a local scale. Flowering of snow‐meadow plants progresses sequentially across local populations along snowmelt gradients, resulting in the restriction of gene flow via pollination process among local populations (i.e., phenological isolation; Hirao & Kudo, 2004, 2008). Because most seeds of *P. multivittatum* are dispersed by gravity around mother plants, long‐distance seed dispersal seems to be rare. Thus, local adaptation of defense strategies may exist in snowy alpine ecosystems. To test this prediction, clarifications of genetic differentiation among populations and the relationship between male flower production and success as a pollen donor under different seed predation situations are required.

## CONFLICT OF INTEREST

We declare that there is no conflict of interest.

## AUTHOR CONTRIBUTIONS

**Gaku Kudo:** Conceptualization (lead); Data curation (equal); Formal analysis (lead); Funding acquisition (lead); Investigation (equal); Methodology (lead); Project administration (lead); Resources (equal); Software (equal); Supervision (lead); Validation (lead); Visualization (equal); Writing‐original draft (lead); Writing‐review & editing (lead). **Akari Shibata:** Conceptualization (supporting); Data curation (equal); Formal analysis (supporting); Funding acquisition (supporting); Investigation (equal); Methodology (supporting); Project administration (supporting); Resources (equal); Software (equal); Supervision (supporting); Validation (supporting); Visualization (equal); Writing‐original draft (supporting); Writing‐review & editing (supporting).

## Data Availability

The data set used for the analyses is archived in Dryad doi: (https://doi.org/10.5061/dryad.b5mkkwhcq).
